# Comprehensive Self-Healing Evaluation of Asphalt Concrete Containing Encapsulated Rejuvenator

**DOI:** 10.3390/ma15103672

**Published:** 2022-05-20

**Authors:** Ali Zain Ul Abadeen, Arshad Hussain, Veerappan Sathish Kumar, Gunasekaran Murali, Nikolai Ivanovich Vatin, Hassan Riaz

**Affiliations:** 1 School of Civil and Environmental Engineering, National University of Sciences and Technology, Islamabad 44000, Pakistan; drarshad@nit.nust.edu.pk (A.H.); hasssan.riaz.khan@gmail.com (H.R.); 2 Faculty of Civil Engineering, Architecture and Geodesy, University of Split, 21000 Split, Croatia; 3 Peter the Great St. Petersburg Polytechnic University, 195251 St. Petersburg, Russia; murali_22984@yahoo.com (G.M.); vatin@mail.ru (N.I.V.)

**Keywords:** self-healing, sodium alginate capsules, asphalt rejuvenator, asphalt, short term ageing, healing index

## Abstract

Ultraviolet radiation, oxidation, temperature, moisture, and traffic loads produce degradation and brittleness in the asphalt pavement. Microcracks develop into macrocracks, which eventually lead to pavement failure. Although asphalt has an inherent capacity for self-healing, it is constricted. As a result, damages build beyond the ability of asphalt to repair themselves. This research employs the in-situ crack healing method of encapsulated rejuvenator technology to enhance the insufficient self-healing capability of roads. This allows the extrinsically induced healing in asphalt to assist it in recovering from damage sustained during service life. Optical microscopy, thermogravimetric analysis, and the compressive load test of capsules were done to characterise their properties. We measured the self-healing behaviour of encapsulated rejuvenator-induced asphalt utilising the three-point bending beam tests on unaged, short-term aged and long-term aged asphalt beams. The rate of oil release before and after healing was quantified using Fourier transform infrared spectroscopy. The results of these tests were utilised to explain the link between healing time, temperature, asphalt ageing, and healing level. Overall, it was determined that the encapsulated rejuvenator was acceptable for mending asphalt mixes because it increased healing temperature and duration, resulting in an up to 80% healing index.

## 1. Introduction

Asphalt is one of the most widely used materials in road construction around the globe. It has alluring properties of cost-effectiveness, noise reduction and exceptional riding quality. Its advantages no doubt benefit society in a lot of ways, but its vulnerabilities, such as harsh environment, continuous traffic, thermal cycles and ultra-violet radiations, result in one of the most critical problems in asphalt, that is, ageing of binder [1,2]. Old asphalt has a higher viscosity and a more rigid structure than new asphalt, indicating its composition has changed dramatically. Surface ravelling, loss of adhesion, cracking and brittleness can all come from these changes in the presence of dampness [3,4]. With ageing, the proportion of asphaltenes in old asphalt grew while the number of aromatics and resins dropped, further exacerbating the ageing process. Aged asphalt’s decreased penetration value, increased viscosity and softening point are obvious signs of diminishing properties [5]. Pavement develops cracks if it is repeatedly loaded at a stress level that is much lower than its ultimate strength. After that, the freezing and thawing cycles may cause even further expansion of the gaps by soaking the water. Damaged pavement repair and reconstruction is a time-consuming and expensive process that adds to asphalt pavement construction’s already significant carbon dioxide gas emissions. It has been claimed in previous research that rejuvenators on pavement surfaces increase pavement life expectancy by many years. However, it has been shown that the rejuvenators did not cure pavement fractures at deeper depths and only acted up to a few millimetres below the surface [6,7].

**Figure 1:**
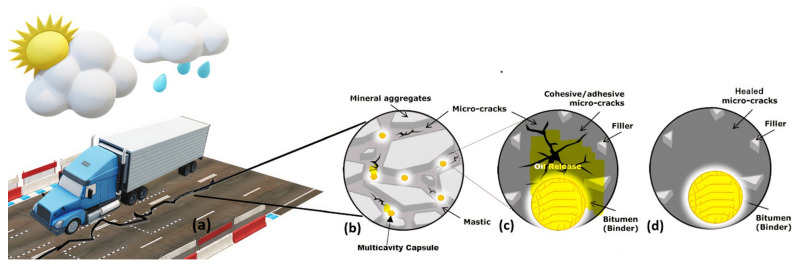
Self-healing of asphalt with encapsulated rejuvenator at different levels. (**a**) Cracks produced in the pavement due to traffic loading and environmental factors; (**b**) multi-cavity capsules carrying rejuvenator present in cracking mixture that result in the partial release of rejuvenator in the crack zone; (**c**) rejuvenator comes out of capsules diffusing into cracks and restoring damaged bitumen properties; (**d**) healed microcracks and rejuvenated bitumen.

Rejuvenators can restore the virgin properties of bitumen and induce volatile compounds to get the original asphaltenes to maltenes ratio back [8,9]. Several vegetable oils and mineral oils have been used by researchers to rejuvenate aged asphalt successfully [10,11,12,13,14,15]. These oils and rejuvenators possess properties to improve the lost properties of bitumen [16,17,18,19,20,21]. Even waste and recycled oils have shown considerable rejuvenating properties [22,23,24,25]. However, rejuvenators may lower shear resistance and pollute the environment when applied to surfaces. Using embedded rejuvenators in asphalt mix has been shown in recent studies to be more helpful in the self-healing of asphalts than using traditional ways of surface rejuvenators [26]. Increasing bitumen flow is essential to fill cracks in the asphalt matrix. Thus, researchers devised in-situ crack healing technologies that improve bitumen drainage into the cracks. The induction or microwave heating included incorporating conducting material in asphalt so that electromagnetic fields could be used to produce heat in the pavement, which caused the bitumen to expand and fill the gaps [27,28]. Figure 1 shows the second technique incorporating rejuvenators packed in shell structure into the asphalt mixture. The volatile compound (oil) leaked out and dispersed throughout the mixture when the shell broke because of traffic load. As a result, the viscosity of bitumen surrounding the capsule decreased, but the oil content remained high, allowing bitumen to flow more freely into the fissures. These volatile compounds are discharged from the capsules, allowing the accompanying bitumen to soften and seal the fissures. There are no re-mixing requirements for the rejuvenated bitumen, as it operates earlier and concentrates on quickening the in-situ fracture healing process in asphalt pavement. Even though the fractures have been repaired, they are also considered to have reduced significantly, and new cracks are prevented from occurring [29,30].

A capsule healing system is an effective way to encapsulate and embed the rejuvenator into an asphalt pavement, i.e., to renew the aged bitumen upon injury and then cure the harm. Rejuvenating agents have been encapsulated in a variety of ways to achieve this goal, including calcium alginate capsules, alginate fibres, prepolymer (melamine-formaldehyde modified by methanol) microcapsules, epoxy microcapsules and nano materials [30,31,32]. Garcia et al. (2011) developed vegetable oil capsules using porous sand as carrier and cement and epoxy as shell [5]. To increase healing at the micro-level, researchers have created micro-capsules using different core and shell materials. Methanol–melamine–formaldehyde-shell and melamine–urea–formaldehyde-shell micro-capsules have shown promising results, with the latter increasing fatigue life by twofold [33,34,35,36]. In general, the micro-capsules improved the asphalt’s fatigue life and healing capability, but formaldehyde used in the shell of micro-capsules is harmful to human health and the environment. Researchers have used novel techniques to create Ca-alginate fibres with many vacuoles loaded with rejuvenators [37,38,39,40,41]. Sodium alginate beads have been used for enhancing the self-healing of asphalt mastic, dense asphalt concrete and stone matrix asphalt and have shown positive impacts on the healing behaviour of the asphalt [29,42,43,44,45]. These showed that sodium alginate capsules release rejuvenator into fractures, and the diffusion starts reinstating properties of the asphalt. In addition, they can deliver larger amounts of the rejuvenator in comparison to micro-sized capsules. According to Norambuena-Contrera et al. (2019) [46], 0.5% of Ca-alginate capsules are best for optimising the mechanical properties of dense asphalt mixes without affecting the rheological qualities.

It is natural for the asphalt binder’s adhesion capacity to deteriorate as the asphalt pavement goes from unaged (UA) to short-term aging (STA) and eventually, long-term aged (LTA) conditions. Researchers have discussed the process of asphalt ageing in length and boiled it down to volatilisation, oxidation and steric hardening [47]. Short-term ageing (STA) accounts for most of the asphalt’s degradation during the construction phase of a new road or highway surface, while long-term ageing (LTA) is the result of traffic loading and environmental factors during service life [48,49]. In the long run, ageing increases the pavement’s low-temperature stiffness and enhances pavement embrittlement, thus making the pavement more prone to cracks [50]. Currently, there is a lack of research on the use of encapsulated rejuvenators to study the combined effect of ageing levels, different healing temperatures and healing durations on the self-healing efficiency of asphalt concrete.

In this research, the effect of the encapsulated rejuvenator (sunflower oil) has been assessed on the healing index on asphalt in changing temperature, ageing level and healing time. To accomplish this, 0.50% capsule percentage was introduced to asphalt mixtures. The properties of capsules were evaluated using surface morphology, thermogravimetric analysis and compressive strength. The crack-healing efficiency of the samples without capsules and with capsules was evaluated at three different ageing levels (UA, STA, and LTA). Four different curing temperatures (20 °C, 25 °C, 30 °C and 40 °C), and nine different healing durations (24 h, 48 h, 72 h, 96 h, 120 h, 144 h, 168 h, 196 h and 216 h) on asphalt concrete samples, with and without capsules using three-point bending tests. The rate of oil release before and after healing was quantified using Fourier transform infrared spectroscopy.

## 2. Materials and Methods

### 2.1. Raw Materials

**Figure 2:**
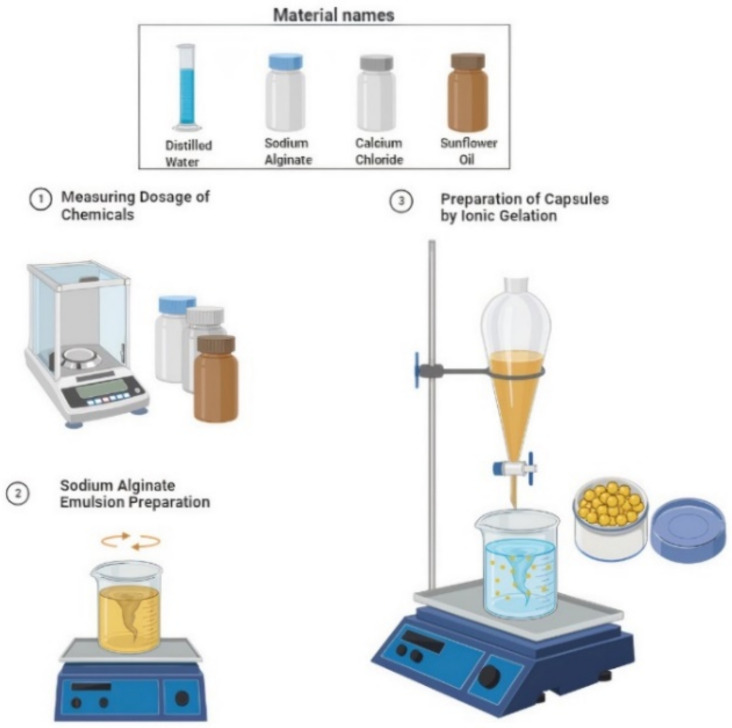
Preparation of Ca-Alginate Capsules.

Bitumen 60/70 was procured from the PARCO refinery with a penetration of 65 (0.1 mm), and a softening point of 49 °C; basalt gravel with a density of 2.725 g/cm^3^ and limestone filler with a density of 2.632 g/cm^3^ was utilised to make the asphalt mixture. The following Table 1 illustrates the National Highway Authority (NHA) B aggregate gradation that is used for aggregate size selection in this research. The rejuvenating agent utilised was sunflower oil, which had a viscosity of 0.282 Pa·s, flashpoint of 225 °C, saturates content of 61.4%, aromatics content of 38.6% and density of 0.933 g/cm^3^ as seen in Table 2. Distilled water was used along with sunflower oil and chemicals for the preparation of C_6_H_9_NaO_7_ emulsion. The capsules’ structure was composed of C_6_H_9_NaO_7_ and CaCl_2_, which were supplied by Daejung Chemicals in the form of powder and granular pellets, respectively, and employed in the capsules’ preparation, as shown in Figure 2.

### 2.2. Capsules Preparation

**Figure 3:**
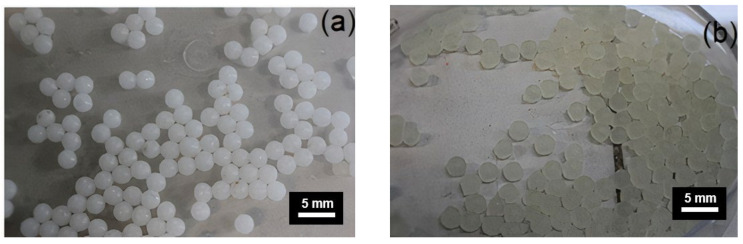
(**a**) Decanted Capsules and (**b**) Dried Capsules.

Encapsulated rejuvenators were produced at 20 °C by preparing sodium alginate emulsion with water and oil at 400 rpm for 10 min. Sodium alginate (2% *w*/*v*), sunflower oil (20 g) and water (80 g) were used for preparation of one batch of emulsion. Calcium Chloride solution (2% *w*/*v*) was prepared in a flask, and C_6_H_9_N_a_O_7_ emulsion was placed in a dropping funnel. The droplets of C_6_H_9_N_a_O_7_ emulsion turned into encapsulated rejuvenator via process of ionic gelation in CaCl_2_ solution as shown in Figure 2. The capsules were collected, decanted and dried for use in the asphalt mixture. Figure 3 depicts the decanted capsules taken out from calcium chloride solution and converted to dried capsules by oven drying them at 40 °C for 24 h. 

### 2.3. Preparation of Asphalt Samples

The NHA B gradation and 60–70 virgin asphalt were utilised in this work to create the asphalt concrete mixtures samples with and without capsules. Table 1 illustrates the NHA B aggregate gradation used in our mixture preparation. Bitumen made up 4.7% of the asphalt mixes, whereas capsules made up 0.5% by weight of the asphalt mixtures. After aggregate sieving and proportioning met the gradation requirements, they were preheated in an oven to 160 °C for four hours. Afterwards, bitumen and aggregate were mixed for 90 s at 160 °C to ensure complete blending of materials. The encapsulated mixture was preprepared by adding dried capsules on the surface of the mixture and swirled for 20 s to ensure they were uniformly distributed throughout the asphalt mixture. STA was done on the loose mix before mixing started, and LTA was conducted on specimens after compaction was conducted. The experimental technique of ageing for the samples was conducted as set forth below in Table 3.

**Figure 4:**
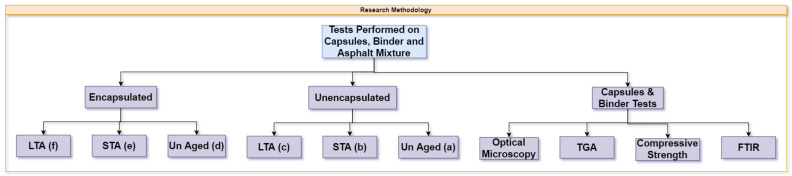
Research methodology.

**Figure 5:**
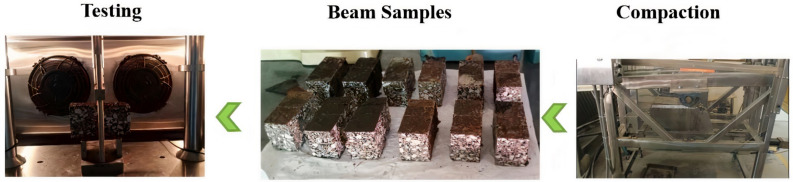
Preparation of Three-Point Bending Beam Samples and UTM Setup.

Asphalt mixture slabs were prepared to utilise the rutting plate moulding machine. Slabs were prepared using a dynamic roller that compacted the asphalt mixture to achieve a 5% air void content. Then, beams measuring 150 mm × 70 mm × 50 mm were cut from slabs, and a notch of 4 mm length and 10 mm height was placed at the bottom of each beam for crack initiation during the three-point bending beam test. These beams were divided into unencapsulated and encapsulated and according to ageing treatment as per Figure 4. Afterwards, beams were taken to the conditioning chamber and processed further according to Figure 5.

### 2.4. Morphological Characterisation

The capsules’ morphology was observed using an optical microscope. To examine the capsules’ internal microstructure, many capsules were split in half and cut to a thickness of around 0.2 mm. The morphological investigation aimed to find out the internal structure of Ca-alginate capsules. This also helped differentiate core–shell structure capsules from multi-cavity capsules that offer multiple healing.

### 2.5. Thermogravimetric Analysis

Asphalt mixing and paving are conducted at high temperatures. This warrants that additives must be carefully selected and tested so that they can endure high-temperature circumstances. Thermogravimetric analysis (TGA) was used to test the high-temperature stability of three different sets of samples i.e., capsules without oil, sunflower oil and capsules with oil. A synchronous thermal analyser was used to conduct the test. The test was performed in a Nitrogen environment and with a heating rate of 10 C/min. TGA measures the mass (%) of capsules with increasing temperature. Three different sets of samples, i.e., capsules without oil, sunflower oil and capsules with oil, were tested to find out thermal stability during the fabrication phase. The mass (%) of the samples was determined at temperatures ranging from ambient temperature to 1000 °C.

### 2.6. Compressive Load Measurement

Capsules had to bear stress and loading during the mixing and compaction phase. Three different sets of capsules in asphalt mixtures, i.e., virgin capsules, STA capsules and LTA capsules, were tested to find out the effect of ageing on the compressive load of capsules. Capsules’ compressive load-taking ability was determined in this investigation using a uniaxial compression test at 25 °C and a loading rate of 0.5 mm/min. When the capsule was broken, a yield point on the stress-displacement curve was formed, and force at the yield point was noted as the ultimate compressive load for the capsule.

### 2.7. Three-Point Bending Beam Test

**Figure 6:**
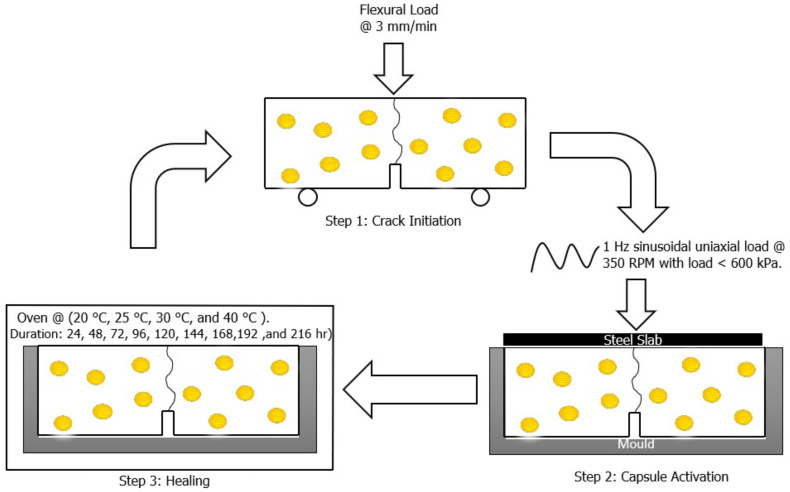
3PB UTM Test Procedure.

Using the three-point bending (3PB) beam test, calcium alginate capsules were evaluated for healing efficacy. To carry out the three PB tests, we used a UTM equipped with a temperature chamber. The experimental setup and settings are shown in Figure 6. Three-point bending beam tests were performed at a low loading speed of 3 mm/s to produce a brittle crack at the notch of the beam following the test setup [54]. It is possible to initiate and propagate cracks into the centre of a specimen during 3PB tests because of the load concentration on the notches.

Three-point bending beam test was used to find the healing index of the asphalt beams for three distinct types of samples: unaged, STA and LTA for encapsulated samples and unencapsulated samples. The stages below describe the procedures for creating cracks, capsule activation, allowing fractures to self-heal and assessing the degree of crack healing. These steps are explained in detail below:

Step 1: Asphalt beam samples were placed in a conditioning chamber at −20 °C for four hours. This was done to ensure the formation of a brittle crack in beams because testing asphalt beams at higher temperatures produces bending in the sample. These asphalt beams were broken at a rate of 3 mm/min, according to step 1 in Figure 6. However, this did not cause many capsules to break and release oil. Then, re-moulded samples were put in a conditioning chamber at 20 °C for two hours. 

Step 2: Capsule activation of beams was done by placing a steel plate on the moulded sample, and the sinusoidal load was applied with specification according to step 2 in Figure 6. After the load application, samples were put in the oven for the healing process according to step 3. 

Step 3: During the healing phase, samples were placed in a temperature-controlled oven at four distinct healing temperatures for nine distinct healing times. After the designated healing time was completed, the asphalt beams were taken out and placed in a conditioning chamber for 2 h at −20 °C. Step 1 was repeated to find the load at which the beam failed again. First flexural load and second flexural load were used to find out the value of healing index of the beam according to Equation (1). This completed the healing cycle of one beam. The same process was repeated for all beam samples in this research. The purpose of testing beams at multiple healing intervals is to find out the optimal healing time that can maximise healing properties. The Healing Index (HI) was used to measure the effectiveness of the healing process, and it was computed as follows:

(1) HI=F1FX

where HI = the healing index (%); F1 = original strength of the sample; Fx = strength after x cycles of healing.

### 2.8. Quantification of Oil Released

Quantification of oil is necessary to understand the amount of oil released in asphalt samples. This helped to understand the rate of oil release before and after the healing process and the link of asphalt ageing with the release of oil ratio (ROR). In the literature, the Fourier-Transform Infrared Spectroscopy (FTIR) test has been used to determine the chemical change in asphalt composition. In this research, the binder samples were extracted from non-encapsulated and encapsulated beams before healing and after healing of asphalt beams. The test was carried out with the help of a spectrum instrument with the wavelength in the range of 400 and 4000 ${\mathrm{cm}}^{-1}$.

**Figure 7:**
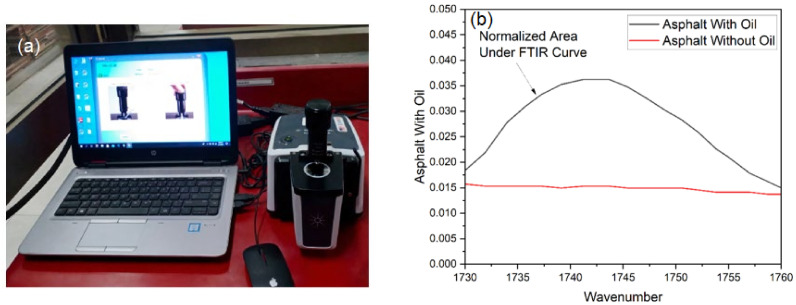
(**a**) FTIR Equipment. (**b**) Graph of asphalt with oil and without oil.

The oil ejected from capsule reakagee may be determined by comparing the peak absorbance at 1700 and 1800 ${\mathrm{cm}}^{-1}$ wavelengths. Between these intervals, bitumen has no peak, but sunflower oil exhibits a definite peak at 1745 ${\mathrm{cm}}^{-1}$ wavelength. The trapezoidal approach of numerical integration is effective for normalising the FTIR absorbance spectra between the wavelengths of 1700 and 1800 ${\mathrm{cm}}^{-1}.$ Figure 7 is an example of a peak absorbance curve of asphalt without and with oil. It is derived from asphalt samples containing 0.5% encapsulated sample by weight of the mixture.

The rate of oil released, ROR, from capsules into the asphalt beams before and after crack-healing experiments was characterised according to the equation used by Norambuena-Contreras et al. (2019) [46].

## 3. Results

### 3.1. Morphological Characteristics of Ca-Alginate Capsules

**Figure 8:**
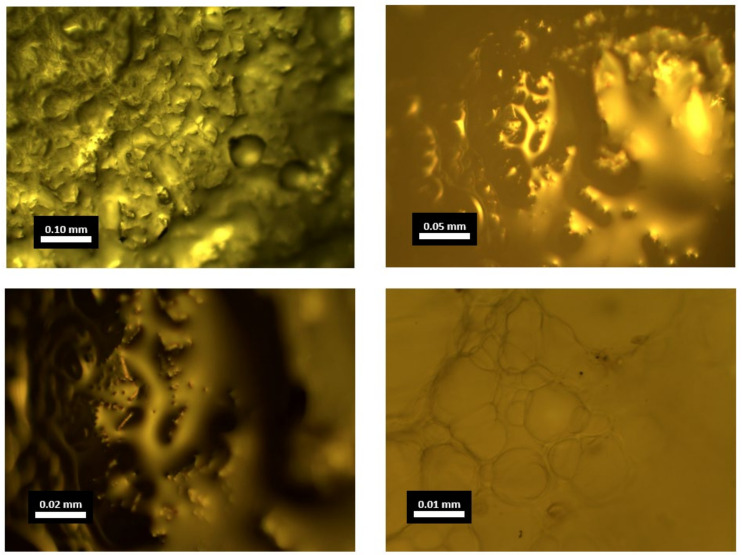
Optical Microscopy of Ca-Alginate Capsules.

OM pictures of capsule cross-sections from 5× to 50× magnification are shown in Figure 8. The internal structure of the capsules has hollow spaces as well as multiple compartments. Thus, its structure can be categorised as multi-cavity rather than the normal core–shell design. Calcium ion cross-linking retained the structure. Normal core–shell capsules rupture upon crack and release rejuvenator, but as their rejuvenator is preserved by a single covering of shell, it has one-time effectiveness. This multi-cavity arrangement allows capsules to release rejuvenator multiple times. It also improves the structural integrity of capsules to resist mixing and compaction stresses. Capsules do not release all the oil when a fracture occurs in this approach, implying that capsules can heal many cracks and long-term repair [29,55]. Figure 8 depicts the cross-section of an individual capsule under an optical microscope with a multi-cavity structure.

### 3.2. Thermal Stability Analysis

**Figure 9:**
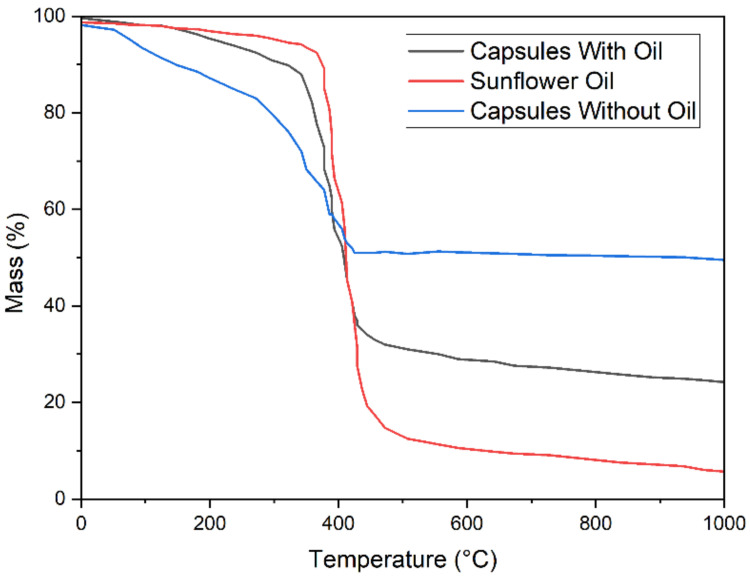
Thermogravimetric Analysis of Ca-Alginate Capsule.

It can be seen in Figure 9 that Ca-alginate capsules could withstand mixing and compaction temperature. There is no significant mass loss below 200 °C, which is promising for the safety of capsules as normally mixing and compaction temperature does not exceed 200 °C. As the temperature rises and crosses 360 °C, mass loss starts to increase dramatically. This shows that capsules remain temperature-resistant until the temperature rises to a level where the oil starts to volatilise, leaving a sodium alginate shell. The leading factor in mass loss is the oil volatilization and loss of –OH group in alginate. The results are consistent with the findings of other researchers [43,44,46,54,55].

### 3.3. Compressive Load of Capsules before and after Ageing

**Figure 10:**
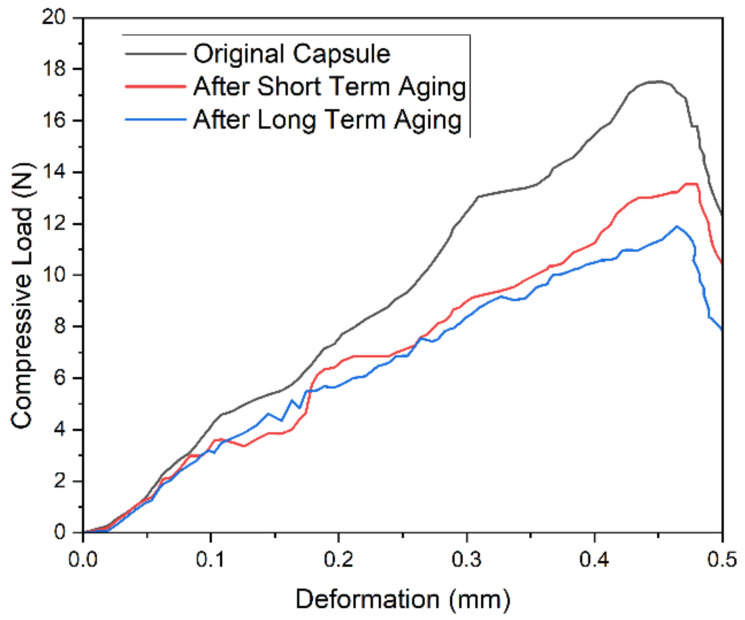
Compressive Load of Capsules.

Figure 10 shows that the sample with original capsules has the highest ultimate compressive load value of 17.5 N in comparison to 13.5 N for short-term aged (STA) capsules and 11.9 N for long-term aged (LTA) capsules. This may be because the ageing of capsules degrades the mechanical properties of capsules. It is observed that the original capsule has higher ultimate load than aged capsules showing that loss of mechanical properties occurs for capsules during the manufacturing of asphalt samples. However, its compressive load is still above the 10 N threshold. Xu et al. (2019) [45] prepared capsules with different alginate/rejuvenator ratios and tested their compressive strength with increasing curing temperature. It showed that increasing temperature resulted in drying of sodium alginate gel and decreased compressive strength of capsules, much like decreased compressive strength in the case of capsules retrieved from STA and LTA beam samples.

### 3.4. Three-Point Bending Test 

**Figure 11:**
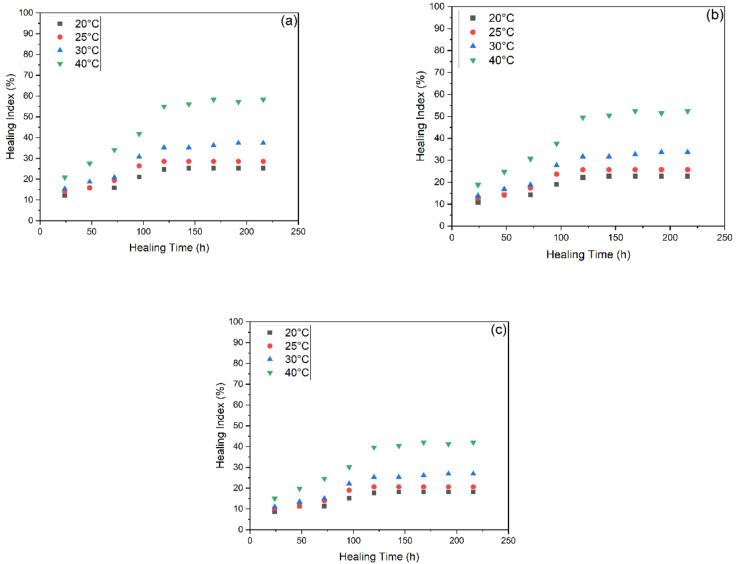
Healing Index of unencapsulated beams with three varying ageing levels after loading: (**a**) unaged, (**b**) short-term ageing (STA), (**c**) long-term ageing (LTA).

**Figure 12:**
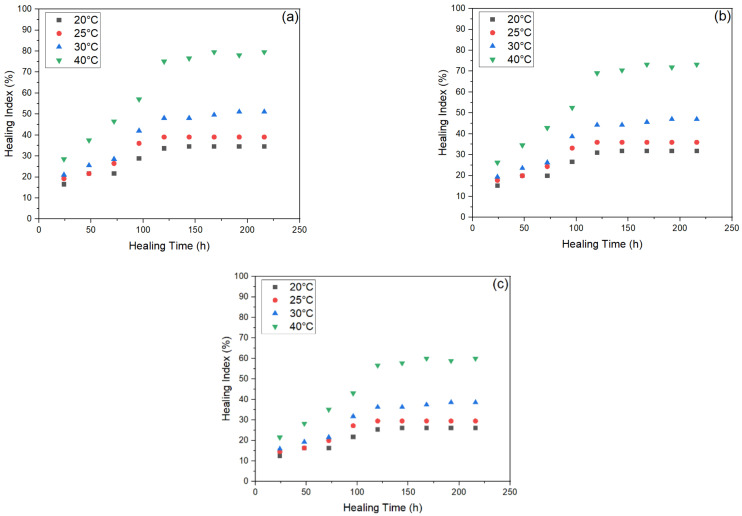
Healing Index of encapsulated beams with three varying ageing levels after loading: (**a**) unaged, (**b**) short-term ageing (STA), (**c**) long-term ageing (LTA).

Figure 11 and Figure 12 illustrate the healing levels obtained by the asphalt samples at four different heating temperature and nine different healing temperatures, with and without capsules. Each bar graph represents a set of beams tested for healing evaluation. The sheer quantity of tests confirms the huge data available for analysis of the healing index of beams. The results of the healing index of unencapsulated and encapsulated asphalt mixture beams subjected to various degrees of ageing treatment are shown in detail. Even without capsules, asphalt beams showed good strength regain due to the bitumen’s inherent healing capability [2]. The ageing treatments stiffen and increase the viscosity of the asphalt binder, reducing the capillary flow of asphalt in the fracture zone. The healing index decreased with STA and LTA because ageing has a detrimental effect on binder chemical properties leading to stiffer, less flowable and decreased healing index. Therefore, the average healing levels for unencapsulated asphalt beams at 20 °C, 25 °C, 30 °C and 40 °C of capsules were 21.19%, 24.30%, 29.70% and 45.47% for unaged, 19.07%, 21.87%, 26.73% and 40.92% for STA and 15.26%, 17.49%, 21.38% and 32.74% for LTA samples, respectively.

Figure 12 shows that encapsulated beams showed superior performance in terms of healing than non-capsulated beams. Therefore, the average healing levels for encapsulated asphalt beams at 20 °C, 25 °C, 30 °C and 40 °C of capsules were 28.93%, 33.17%, 40.54% and 62.06% for unaged, 26.61%, 30.52%, 37.30% and 57.10% for STA and 21.82%, 25.02%, 30.58% and 46.82% for LTA samples, respectively. The healing index of encapsulated beams of unaged, STA and LTA beams exceeded those of unencapsulated beams. Ageing of asphalt binder during the fabrication process is a natural phenomenon, but the release of oil through capsule pores even before the three-point bending beam test renews the properties of the aged binder to some degree. This is the reason for the relative retention of the healing index for STA compared to LTA. However, this effect fades away as for LTA oil produced during ageing treatment was insufficient to counterbalance the detrimental impact of ageing entirely. Thus, a clear decrease is seen in the healing index as ageing progresses.

**Figure 13:**
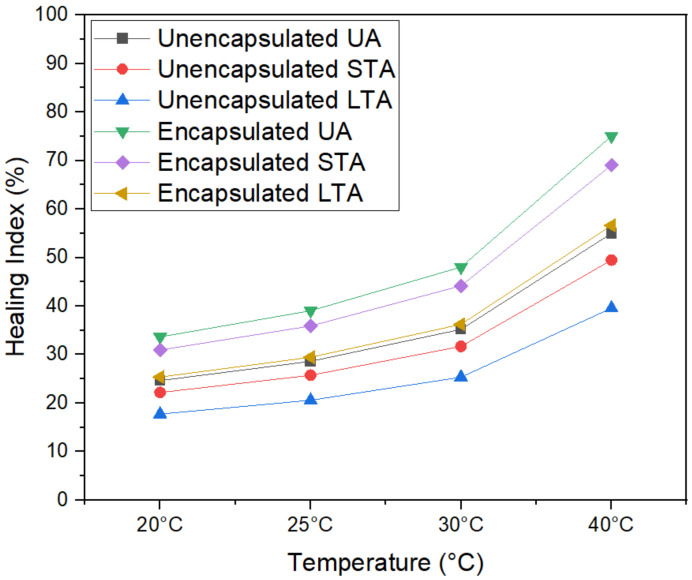
Healing Index of unencapsulated and encapsulated beams at 120 h with three varying ageing levels after loading.

**Figure 14:**
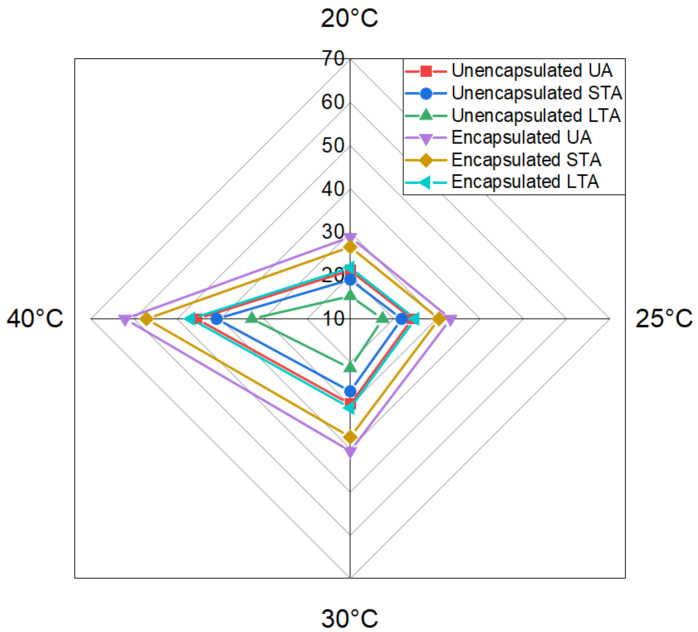
Radar chart of average healing index of encapsulated and unencapsulated beams with three varying ageing levels after loading.

Figure 13 shows the healing index of all six types of samples at 120 h of healing duration with relation to different temperatures. The ageing process applied to asphalt concrete causes capsules to age, decreasing the oil release rate. Additionally, the ageing operation would age the spilled oil, diminishing the ability of old asphalt to restore itself. Capsules added to asphalt concrete with varying degrees of ageing treatment diminish the healing index of test beams without and with capsules. These results explain the rejuvenator release, healing time and temperature relationship as seen in Figure 14. As per the effect of increasing temperature on healing, as the healing temperature increases, the flowability of bitumen naturally increases and crack closure becomes easier. This results in improved healing performance with increasing temperature. Secondly, healing time has a diverse range in this test. This is consistent with the finding of researchers that found that increase in healing duration has positive correlation with crack healing [43,46]. The healing index increases until 120 h, then it starts to flatten out. This is because higher temperature and higher healing time increase the probability of potential released oil and restoration potential. However, it shows that after 120 h, further healing does not produce much effect on the healing index of samples. That might be because increasing the healing duration by more than 120 h does not help in further diffusion and crack closure.

### 3.5. Oil Quantification Test

**Figure 15:**
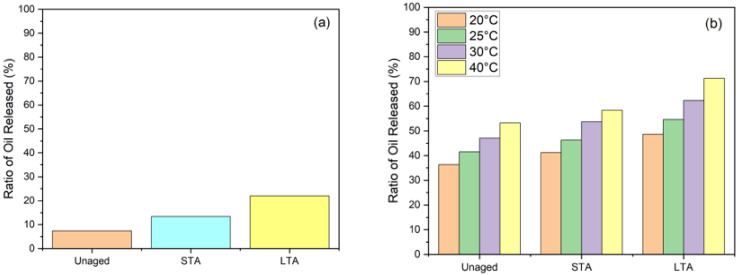
Quantification of oil released: (**a**) before healing (**b**) after healing.

Figure 13 depicts the oil released (ROR) ratio of capsules, which was used to quantify the oil released in asphalt mixture manufacture. This process has been divided into two phases, i.e., (a) before healing and (b) after healing. Before healing, the ROR of capsules for unaged asphalt mixture was 7.45%. The ROR of capsules with short-term ageing treatment in loose asphalt mixtures was 13.4% after mixing before compaction and 22.1% after compaction, implying that the capsules would release more oil inside after LTA in asphalt mixtures. The release of oil from capsules because of short-term ageing may be able to mitigate the detrimental impacts of ageing on the asphalt properties. It was found that ROR increased as healing time and temperature were increased after testing. Figure 15 shows the average ROR for asphalt mixtures with capsules at 20 °C, 25 °C, 30 °C and 40 °C of capsules were 36.43%, 41.54%, 47.13% and 53.27% for unaged, 41.22%, 46.35%, 53.78% and 58.41% for STA and 48.67%, 54.65%, 62.39% and 71.22% for LTA samples, respectively

It is envisaged that the encapsulated oil would leak out of the capsules during construction, hence reducing the amount to which the asphalt mixture ages due to thermal oxides during mixing and compaction. The oil released before healing is substantially low, indicating that maximum capsule breakage was done during the healing phase. Although the oil release of capsules inside aged asphalt concrete increased with ageing, its impact on increasing healing index decreased due to loss of volatiles from binder and ageing of oil. Due to sunflower oil’s in-situ rejuvenation, the released oil may be able to mitigate some of the detrimental effects of ageing therapy on the asphalt by offsetting some of the negative consequences.

## 4. Conclusions

This research characterises and evaluates the influence of calcium alginate capsules on the self-healing capacity of asphalt mixes, taking into consideration the ageing of the mixtures. To revitalise the old asphalt binder by restoring the light components that have been lost, calcium alginate capsules are used as a key means of doing so. Ca-Alginate capsules containing sunflower oil were prepared via ionotropic gelation.

1. The morphology of capsules was seen through optical microscopy, and it was found that capsules had multiple compartmentalised insides. Thus, their structure can be categorised as multi-cavity rather than the normal core–shell design. Capsules were subjected to thermogravimetric analysis to ensure the thermal stability of capsules. Results proved that all capsules could resist the mixing temperature of asphalt, and oil loss never exceeded 5% under 200 °C. Ca-alginate capsules showed good mechanical performance, with original capsules having the highest value of 17.5 N in comparison to 13.5 N of short-term aged (STA) capsules and 11.9 N for long-term aged (LTA) capsules.
2. Encapsulated beams showed superior performance in terms of healing than non-capsulated beams. The average healing levels for asphalt mixtures without capsules at 20 °C, 25 °C, 30 °C and 40 °C of capsules were 28.93%, 33.17%, 40.54% and 62.06% for unaged, 26.61%, 30.52%, 37.30% and 57.10% for STA and 21.82%, 25.02%, 30.58% and 46.82% for LTA samples, respectively. On the other hand, the average healing levels for asphalt mixtures with capsules at 20 °C, 25 °C, 30 °C and 40 °C of capsules were 28.93%, 33.17%, 40.54% and 62.06% for unaged, 26.61%, 30.52%, 37.30% and 57.10% for STA and 21.82%, 25.02%, 30.58% and 46.82% for LTA samples, respectively.
3. The healing index of encapsulated beams of unaged, short-term aged, and long-term aged beams exceeded those of asphalt mixture beams without capsules. It was proven that higher temperature and higher healing time had a positive correlation to the healing index while ageing has negative correlation with the healing index. Thus, 120 h of healing time can be considered the best performing asphalt self-healing environment as a further increment in healing time resulted in the same healing index. Thus, embedded rejuvenators can successfully be used in asphalt pavements to improve crack healing performance.
4. Before healing, the ROR of capsules for unaged asphalt mixture was 7.4%, 13.4% for short-term ageing treatment in loose asphalt mixtures and 22.1% for long-term ageing. FTIR test confirmed the release of oil during the three-point bending beam test as the average ROR for asphalt mixtures with capsules at 20 °C, 25 °C, 30 °C and 40 °C of capsules were 36.43%, 41.54%, 47.13% and 53.27% for unaged, 41.22%, 46.35%, 53.78% and 58.41% for STA and 48.67%, 54.65%, 62.39% and 71.22% for LTA samples, respectively.

## Figures and Tables

**Table 1:** Aggregate gradation NHA Class B.

Sieve Diameter (mm)	NHA B Lower Limit (%)	NHA Upper Limit (%)	Size Chosen (%)
19	100	62	100
12.5	75	90	82.5
9.5	60	80	70
4.75	40	60	50
2.38	20	40	30
1.18	5	15	10
0.075	3	8	5.5

**Table 2:** Properties of Sunflower Oil.

Viscosity (Pa·s (60 °C))	Flash Point (°C)	Saturates (%)	Aromatics (%)	Density g/cm^3^ (15 °C)
0.282	225	61.4	38.6	0.933

**Table 3:** Short term ageing and long-term ageing for testing.

Ageing Type	Method	Temperature (°C)	Time (h)
Short term ageing [37,39,51,52,53,54]	Distributed loosely on a tray in the oven	135	4
Long Term Ageing [37,39,51,52,53,54,55]	Forced ventilation in oven	85	120

## Data Availability

Not applicable.
